# Decitabine mildly attenuates *MLL*‐rearranged acute lymphoblastic leukemia in vivo, and represents a poor chemo‐sensitizer

**DOI:** 10.1002/jha2.81

**Published:** 2020-08-24

**Authors:** Pauline Schneider, Patricia Garrido Castro, Sandra M. Pinhanços, Mark Kerstjens, Eddy H. van Roon, Anke H.W. Essing, M. Emmy M. Dolman, Jan J. Molenaar, Rob Pieters, Ronald W. Stam

**Affiliations:** ^1^ Princess Máxima Center for Pediatric Oncology Utrecht The Netherlands; ^2^ Department of Pediatric Hematology/Oncology Erasmus MC ‐ Sophia Children's Hospital Rotterdam The Netherlands

**Keywords:** acute lymphoblastic leukemia, chemo‐sensitizer, decitabine, DNA demethylating agent, *KMT2A*, *MLL*, xenograft mouse models

## Abstract

*MLL*‐rearranged acute lymphoblastic leukemia (ALL) represents a highly aggressive ALL subtype, characterized by aberrant DNA methylation patterns. DNA methyltransferase inhibitors, such as decitabine have previously been demonstrated to be effective in eradicating *MLL*‐rearranged ALL cells in vitro.

Here, we assessed the in vivo anti‐leukemic potential of low‐dose DNA methyltransferase inhibitor decitabine using a xenograft mouse model of human *MLL*‐rearranged ALL. Furthermore, we explored whether prolonged exposure to low‐dose decitabine could chemo‐sensitize *MLL*‐rearranged ALL cells toward conventional chemotherapy as well as other known epigenetic‐based and anti‐neoplastic compounds.

Our data reveal that decitabine prolonged survival in xenograft mice of *MLL*‐rearranged ALL by 8.5 days (*P* = .0181), but eventually was insufficient to prevent leukemia out‐growth, based on the examination of the MLLAF4 cell line SEM. Furthermore, we observe that prolonged pretreatment of low‐dose decitabine mildly sensitized toward the conventional drugs prednisolone, vincristine, daunorubicin, asparaginase, and cytarabine in a panel of *MLL*‐rearranged cell lines. Additionally, we assessed synergistic effects of decitabine with other epigenetic‐based or anticancer drugs using high‐throughput drug library screens. Validation of the top hits, including histone deacetylase inhibitor panobinostat, BCL2 inhibitor Venetoclax, MEK inhibitor pimasertib, and receptor tyrosine kinase foretinib, revealed additive and moderate synergistic effects for the combination of each drug together with decitabine in a cell line‐dependent manner.

## 1 INTRODUCTION

Rearrangement of the *Mixed Lineage Leukemia (MLL*, or *KMT2A)* gene is a cytogenetic aberration highly prevalent in infants (<1 year of age) diagnosed with acute lymphoblastic leukemia (ALL), where it constitutes ∼80% of the cases. *MLL* rearrangements mark a very aggressive ALL subtype. Despite highly intensified treatment protocols, event‐free survival (EFS) chances for *MLL*‐rearranged infant ALL only reach 35‐40%, falling well short of survival rates of infants and older children with ALL carrying other cytogenetic aberrations (70‐90%) [1, 2, 3]. Hence, novel treatment strategies based on the specific molecular pathobiology are crucial.

The main oncogenic hit of *MLL*‐rearranged ALL is the in‐frame fusion of the *MLL* gene with one of multiple fusion partner genes, generating *MLL* fusion genes that encode chimeric proteins that drive leukemogenicity and disease maintenance [4, 5, 6]. MLL itself functions as a histone methyltransferase, and the most recurrent fusion partner genes, *AF4* (*AFF1*), *ENL* (*MLLT1*), and *AF9* (*MLLT3*), all encode proteins that are part of complexes regulating epigenetic mechanisms. As truncated parts of the MLL fusions these proteins interfere with and mistarget the regulating complexes, hijacking their activities [7]. As a result, *MLL‐*rearranged acute leukemia typically presents with a highly abnormal epigenome, reflected by aberrant DNA methylation patterns [8, 9, 10] and histone modification signatures [11], which alter the epigenetic and transcriptomic landscape of the cell. Consequently, several epigenetic drug classes, including DOT1L histone methyltransferase, BET protein, and histone deacetylases (HDAC) inhibitors, have shown promising results in *MLL*‐rearranged ALL animal models [12, 13, 14, 15, 16], providing preclinical rationales for their implementation in current and future clinical trials [17].

However, despite their known cytotoxicity against *MLL*‐rearranged ALL cells in vitro [7, 10, 17, 18], preclinical in vivo activity studies of another pivotal class of epigenetic drugs, that is, the DNA methyltransferase inhibitors (DNMTi), such as decitabine and 5‐azacytidine, are limited. Therefore we assessed the in vivo anti‐leukemic potential of low and clinically relevant dosages of decitabine for a prolonged timespan in a *MLL*‐rearranged ALL xenograft mouse model. Furthermore, using high‐throughput combinatorial drug library screens, we explored whether prolonged low‐dose decitabine would epigenetically prime and chemo‐sensitize *MLL*‐rearranged ALL cells toward standard chemotherapy, as well as toward an array of other, mostly FDA‐approved compounds.

## 2 MATERIALS AND METHODS

### 2.1 Animal models

Animal experiments were performed under compliance of Dutch legislation after approval of the institutional Animal Ethics Committee at the Erasmus MC, Rotterdam, the Netherlands. Immunodeficient NOD.Cg‐*Prkdc^scid^Il2rg^tm1Wjl^*/SzJ (NSG) mice (n = 26) were transplanted intrafemurally (i.f.) with the luciferase‐expressing *MLL*‐rearranged ALL reporter cell line SEM‐SLIEW (10^5^ cells per mouse). Mice were kept in individually ventilated cages with food and water ad libitum. Bioluminescence measurements were performed under isoflurane narcotization to confirm engraftment 3 days post‐injection and to monitor disease progression every other week; RediJect d‐Luciferin (PerkinElmer) substrate was administered intraperitoneally and bioluminescence signals were visualized and whole‐body photon flux (photons/sec) quantified on an IVIS Spectrum system using Living Image software (PerkinElmer). To overcome the therapeutic limitations of the short physiological half‐life of decitabine in vivo, the cytidine deaminase inhibitor tetrahydourine (THU, Sigma‐Aldrich, 4 mg/kg in saline) was administered i.p. in parallel, on the opposite abdominal quadrant. The control group was treated with the corresponding vehicle (10% DMSO in saline).

Mice showing overt clinical signs of leukemia and reaching humane end points as indicated by Animal Ethical Committee statutes and in compliance with ARRIVE guidelines (lethargy, acute weight loss > 15%, severe behavioral abnormalities, hind limb paralysis, etc) were humanely culled, and systemic leukemic burden was determined using multicolor flow cytometry, as described before [15]. Statistical significance was determined by log‐rank testing.

### 2.2 Cell culture

The *MLL*‐rearranged B‐cell precursor acute lymphoblastic leukemia (pre‐B ALL) cell lines SEM (MLL‐AF4^+^) and KOPN‐8 (MLL‐ENL^+^) were purchased from DSMZ (Braunschweig, Germany), while the *MLL*‐rearranged ALL cell line ALLPO (MLL‐AF4^+^) was a kind gift from the lab of Dr. Cazzaniga, University of Milano‐Bicocca, Italy. SEM‐SLIEW is derived from MLL‐AF4+ cell line SEM and was modified to express the luciferase reporter gene [15].

All cell lines were cultured in Gibco™ RPMI‐1640 with GlutaMAX™, supplemented with 10% fetal calf serum, 100 IU/mL penicillin, 100 IU/mL streptomycin, and 0.125 μg/mL amphotericin B (Thermo Fisher Scientific, Waltham, USA) at 37°C under 5% CO_2_ atmosphere. Cell line integrity was regularly checked by DNA fingerprinting as well as mycoplasma free status by mycoplasma testing.

### 2.3 High‐throughput drug screening

The *MLL*‐rearranged ALL cell lines SEM was pretreated for 14 days with 10 nM decitabine (5‐Aza‐2′‐deoxycytidine, Merck, Sigma‐Aldrich, St. Louis, USA) or equal amount of DMSO (vehicle), compound‐containing medium was refreshed every 2 days and cells passaged every 4 days. Subsequently the pretreated cells were tested on a drug library containing all 43 compounds of the Enzo SCREEN‐WELL® epigenetics library (Enzo Life Sciences, Farmingdale, USA), all 59 compounds of the Cayman epigenetic library (Cayman chemicals, Ann Arbor, USA), all 157 compounds of the Sequoia anti‐neoplastic drug library (Sequoia Research Products, Pangbourne, UK), 84 FDA approved compounds of interest (Spectrum, MicroSource, Gaylordsville, USA), as well as 26 additional compounds of interest (Sigma‐Aldrich, Selleckchem. All compounds tested are listed in Table S1.

The decitabine or vehicle pre‐treated cell lines were seeded in 384‐well plates at 10 000 cells/well and treated with 10, 100, or 1000 nM of the compounds using the Sciclone ALH 3000 liquid handling robot (Perkin Elmer). Control samples were treated with DMSO (maximum concentration 0.5% v/v). The cell viability was assessed by a 4‐day thiazolyl blue tetrazolium bromide (MTT; Sigma) assay as previously described [19]. The cell viability was normalized to the DMSO controls. This normalized cell viability of the three concentrations of each compound was used to calculate the area under the curve (AUC) for the compound using GraphPrism. The top hits were defined as drugs with a reduction of more than 30% AUC in the decitabine pretreated SEM cells compared to vehicle‐treated cells.

### 2.4 Drug exposures and synergy determination

For the validation of the top hits from the high‐throughput drug screen and synergy studies, expanded dose response curves were made using the Tecan D300 Digital Dispenser (Tecan, Switzerland) to dispense the drug. Again the drug response on the cell viability was assessed by a MTT assay. MTT data were normalized to DMSO control, tolerating a maximum concentration of ≤0.5% (v/v). Experiments performed in triplicate for ALLPO and SEM, in duplo for KOPN8, with three technical replicates each.

Drug synergy between decitabine and the combined compounds was determined using BLISS independence model calculations [20], with the equation: $E_{combi}=E_{A}\;+E_{B}-E_{A}*E_{B}$, where E_A_ represents the fraction of inhibition by drug A alone at a specific concentration, and E_B_ represents the fraction of inhibition by drug B alone. The excess over Bliss (EOB) is the difference between the Bliss expectation and the observed growth inhibition of the combination of A and B (E_combi_) at a given dosage. The percentage excess over Bliss (%EOB) was calculated by multiplying the EOB by 100%. A positive %EOB indicates an additive or synergistic effect, while a negative score indicates an antagonistic effect. Synergy was defined if the inhibition of the combination (E_combi_) showed an excess over BLISS of >10%, while antagonism was defined if the *E*_combi_ showed an excess over BLISS of <−10%.

### 2.5 Western blotting

Cell pellets of the cell lines pretreated with decitabine or vehicle were collected at several time points and lysed with RIPA buffer supplemented with protease inhibitors (Thermo Fisher Scientific, Waltham, USA). Western blot analysis was performed for two independent drug exposure experiments. Twenty‐five microgram of whole cell protein lysates were resolved on 10% polyacrylamide Mini‐PROTEAN^®^ TGX™ Precast Gels (Bio‐Rad, Hercules, USA), and subsequently transferred to nitrocellulose membranes using the Transblot Turbo Transfer System (Bio‐Rad, Hercules, USA). Membranes were blocked with 5% skim milk in TBS and probed with primary antibodies against rabbit polyclonal anti‐DNMT1 (1/1000 dilution, #M0231S, New England Biolabs, Ipswich, MA, USA). Anti‐GAPDH rabbit monoclonal antibodies (1/1000 dilution, # 2118, Cell Signaling Technology Inc., Danvers, USA) were used to detect GAPDH and confirm equal loading in all lanes. The membranes were then probed with infrared‐labeled secondary antibodies IRDye 800CW goat‐anti‐rabbit antibody (1/2000 dilution, #926‐32211, LI‐COR, Lincoln, USA) and IRDye 680 goat‐anti‐mouse antibody (1/2000 dilution, #926‐32220, (LI‐COR, Lincoln, USA). Images were acquired using an Odyssey Infrared Imaging System (LI‐COR, Leusden, the Netherlands) and protein expression was quantified using the Odyssey software Image Studio Lite version 4.0.

## 3 RESULTS

### 3.1 Decitabine monotherapy mildly attenuates leukemia progression in MLL‐rearranged ALL xenografts

**FIGURE 1:**
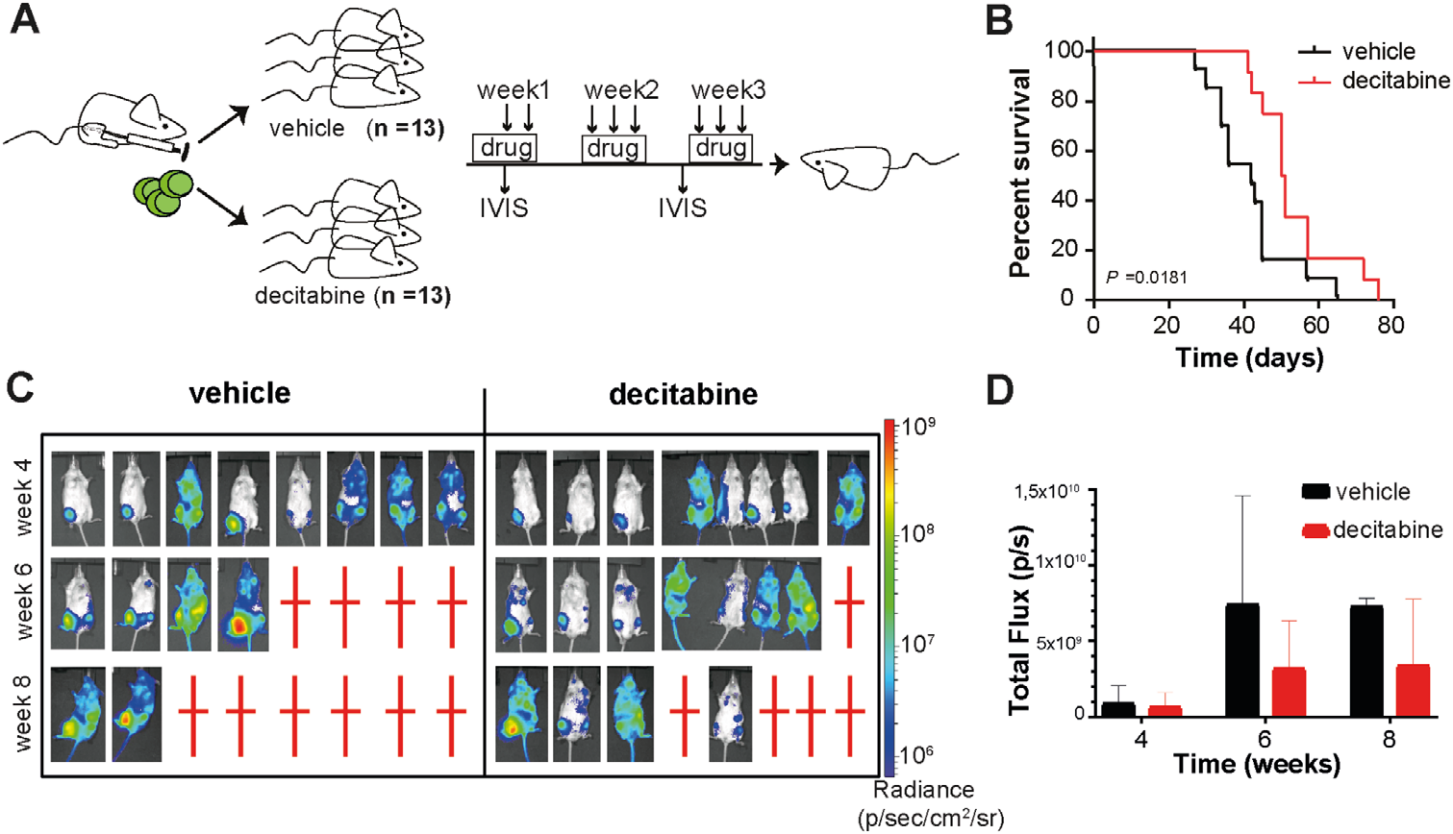
Decitabine mildly attenuates *MLL*‐rearranged ALL disease progression in xenograft mouse models. Experimental design: NSG mice (n = 26) were injected with 10^5^ SEM‐SLIEW cells. Three days post‐transplantation, mice were imaged and randomly allocated to different treatment arms; treated with either 0,1 mg/kg decitabine + 4 mg/kg THU or vehicle, 10% DMSO in saline (A). Kaplan‐Meyer plots illustrate a significantly extended median survival in the decitabine‐treated group (n = 13, 50.5 days) compared to controls (n = 13, 42 days). Statistical significance was determined by log‐rank testing (B). Longitudinal intra‐vital bioluminescence imaging of a representative panel of mice showed confirmed an overall reduced systemic disease burden in decitabine‐treated mice compared to controls. Red crosses represent deceased mice (C). Quantification of intra‐vital imaging data of each individual mouse from the vehicle and decitabine groups (black and red, respectively), as measured on weeks 4, 6, and 8. Data are presented as mean photonic flux with standard deviation (ie, the number of emitted photons per second) with (D)

Xenograft mouse models still represent the standard for in vivo anti‐leukemic drug efficacy testing in *MLL*‐rearranged ALL, as to date bona fide genetic mouse models have not yet been reliably established for this type of leukemia. In order to generate xenografts, we used a previously described reporter cell line, SEM‐SLIEW, which is derived from the MLL‐AF4‐positive B‐cell precursor ALL cell line SEM. SEM‐SLIEW was modified to express the luciferase reporter gene, allowing for longitudinal in vivo disease monitoring by bioluminescence [15]. Decitabine dose response curves showed comparable sensitivity of SEM‐SLIEW and its parental cell line SEM to the drug in vitro (Figure S1). Xenografts were established by injecting 10^5^ SEM‐SLIEW cells intrafemurally into the bone marrow of immunodeficient NSG mice, creating an orthotopic model. Successful engraftment was confirmed by bioluminescence post‐transplantation, and mice were divided into a control (n = 13) and treatment group (n = 13). The treatment group was intraperitoneally injected with a low dose of decitabine (0.1 mg/kg), three times a week. The control group was injected with the corresponding vehicle (10% DMSO in saline) (Figure 1A). One of the therapeutic limitations of decitabine in vivo is its short physiological half‐life due to metabolization by liver cytidine deaminases [21]. As previous animal studies have shown that co‐administration of tetrahydourine (THU), a cytidine deaminase inhibitor, elevates decitabine plasma levels 10‐fold, while revealing no anti‐leukemic efficacy in monotherapy, we co‐injected decitabine with THU (4 mg/kg in saline) [22, 23, 24, 25, 26, 27].

Leukemia progression was assessed by bioluminescence imaging every other week, and mice displaying overt signs of leukemia were sacrificed. The median survival times were 50.5 days in the decitabine‐treated mice and 42 days in the control mice revealing a prolonged survival in the treated mice of 8.5 days (*P* = .0181, Figure 1B). The disease burden was reduced as illustrated by whole body luminescence measurements (Figure 1C,D). Although decitabine prolonged survival in the xenograft mouse model of *MLL* rearranged ALL, decitabine was insufficient to prevent leukemia out‐growth.

Previous reports hinted on the use of decitabine as a chemo‐sensitizer in a variety of cancer types [28, 29, 30]. Hence, to elucidate whether decitabine would exert chemo‐sensitizing effects in *MLL*‐rearranged ALL, we next performed high‐throughput combinatorial drug screens.

### 3.2 Chemo‐sensitizing effect of decitabine toward conventional chemotherapeutics in *MLL*‐rearranged ALL

**FIGURE 2:**
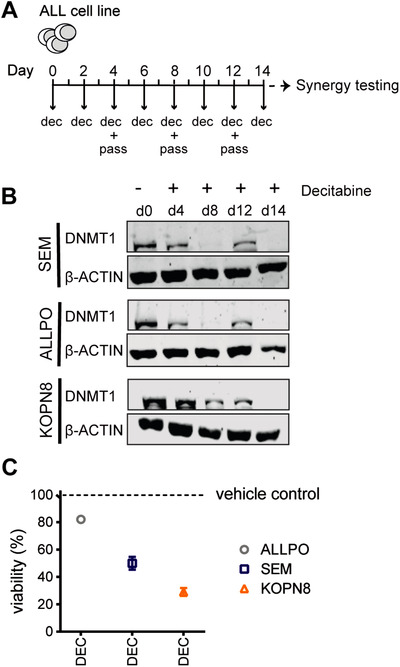
Setup of combinatorial synergy screening and effect of decitabine monotherapy on cell lines. Schematic setup in vitro synergy testing of decitabine: the *MLL*‐rearranged ALL cell lines SEM, ALLPO, and KOPN8 were pre‐treated for 14 days with 5 nM decitabine or vehicle and subsequently exposed to synergy testing (A), the effect on DNMT1 protein expression was determined using western blotting with β‐ACTIN as a loading control (B). Mean percentage of cell viability effects of 14 days of 5 nM decitabine (DEC) pretreatment or vehicle control in *MLL*‐rearranged ALL cell lines SEM, ALLPO, and KOPN8 with standard deviation. Viability was determined using MTT assays. Data has been corrected for the effect of vehicle (C)

The chemo‐sensitizing capability of decitabine was assessed by performing a combinatorial screen of decitabine with prednisone, asparaginase, cytarabine, daunorubicin, or vincristine, which represent cornerstone drugs in current *MLL*‐rearranged infant ALL treatment [2, 3]. Prior to synergy testing, the *MLL*‐rearranged ALL cell lines SEM, ALLPO, and KOPN8 were first pretreated with a low dose of 5 nM decitabine or corresponding vehicle (controls). Since high concentrations of decitabine cause DNA damage by the formation of DNA double strand breaks [31, 32], we used a low‐dose decitabine similar to others [33] to solely evaluate the demethylating effect of decitabine. The low dose of decitabine is clinically relevant, since in pediatric patients with acute myeloid leukemia, a dosage of 20 mg/m^2^ decitabine is safely achievable [34, 35], leading to overall maximal plasma levels of 100 ng/mL or 0.4 μM, which will decrease substantially within 1 h. As DNMT inhibitors typically require several cell divisions to fully exert their demethylating activity, decitabine pretreatment was performed using a prolonged period of exposure of 14 days [36]. Due to the short half‐life of decitabine, the drug was refreshed every other day, and passaging of the cells was performed every 4 days for optimal cell growth conditions (Figure 2A).

The demethylating effect of decitabine during 14 days of pretreatment was assessed by monitoring DNMT1 protein expression. Decitabine represents a deoxycytidine analogue that, like normal deoxycytidines, becomes incorporated into the DNA during replication. Once incorporated, decitabine covalently binds and traps DNA methyltransferases (DNMTs), thereby depleting subsequent daughter cells from functional DNMTs in consecutive cell cycles [37]. Depletion of DNMT1 is commonly used as a reliable read‐out for DNA demethylation, and we confirmed that expression of DNMT1 was completely lost after the 14‐day pretreatment with low‐dose decitabine in all cell lines tested (Figure 2B). In KOPN8 reduction of DNMT1 expression is seen after 4 days and further reduced in the later timepoints until a total loss after 14 days. In SEM and ALLPO inhibition of DNMT1 expression is evident after 4 days and completely lost after 8 days. In these cell lines, the band for DNMT1 reappears after 12 days, probably due to decay of decitabine. Interestingly, all three *MLL*‐rearranged ALL cell lines displayed different responses in viability to the 14‐day low‐dose decitabine pretreatment. The low concentration of 5 nM of decitabine corresponded to the IC_80_, IC_50_, and IC_30_ values in ALL‐PO, SEM, and KOPN8, respectively (Figure 2C).

**FIGURE 3:**
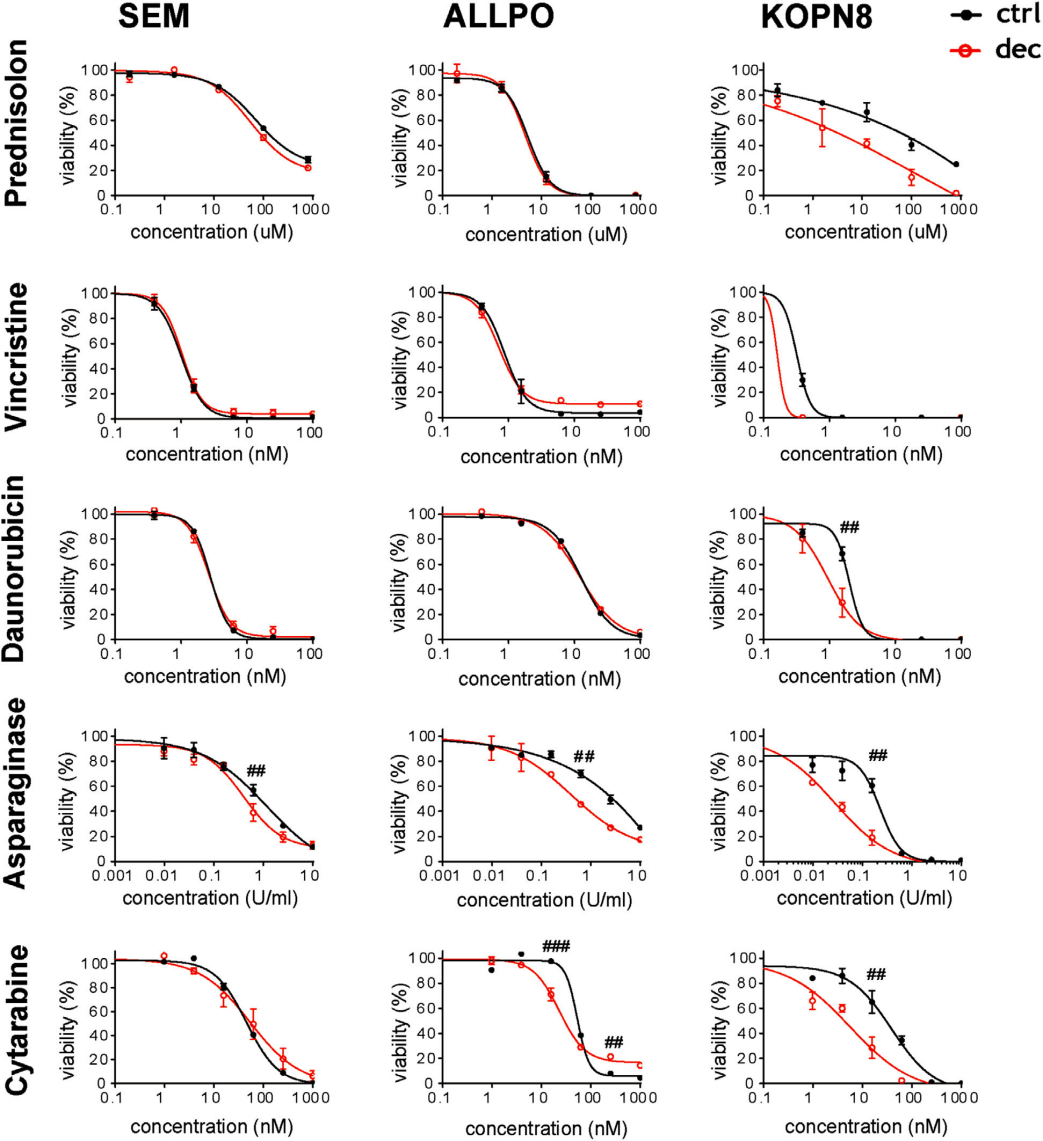
Long‐term low‐dose decitabine treatment acts as a poor chemo‐sensitizer in *MLL*‐rearranged ALL cells. Chemo‐sensitizing effect of decitabine pre‐treatment (dec) on current chemotherapeutics. Decitabine pretreated cells were subsequently cultured with additional compounds in the presence or absence (ctrl) of the hypomethylating agent for 4 additional days. Synergy is determined using Bliss independence model. Percentage excess over Bliss (EoB) is indicated: ^##^EoB > 10%; ^###^EoB > 20%. Error bars represent the standard error of the mean (SEM). Graphs represent the average of n = 3 independent experiments (n = 2 for KOPN8)

After the 14‐day period of decitabine pretreatment, the leukemic cells were subjected to synergy testing, using 5 nM decitabine in combination with prednisone, asparaginase, cytarabine, daunorubicin, or vincristine, which are currently used in the treatment of *MLL*‐rearranged infant ALL. The in vitro efficacy of each drug combination was assessed by 4‐day dose‐response curves (MTT assays), normalized to the effects of decitabine as a single agent and analyzed for synergistic, additive, or antagonistic effects by means of the Bliss Independence model [20]. For all three tested cell lines, a mild chemo‐sensitizing effect was observed toward asparaginase in the decitabine treated cells (Figure 3). The combination of decitabine and 15.6 nM cytarabine appeared to have a synergistic effect in ALLPO, while showing an antagonistic effect for the combination of decitabine and 250 nM of cytarabine. Enhanced sensitivity toward all the conventional drugs was evident in KOPN8, although this is not considered as synergy by the Bliss independence model due to the increased effect of decitabine alone reducing cell viability by 70% (Figure 2C). Taken together we observe decitabine chemo‐sensitizes toward the conventional drugs prednisolone, vincristine, daunorubicin, asparaginase, and cytarabine for inhibition of cell survival of *MLL* rearranged ALL in a limited range of concentrations. Additionally, the observed synergy was not consistent in all of the cell lines tested. These results indicate prolonged exposure to low‐dose decitabine is sufficient to completely deplete *MLL*‐rearranged ALL cells from DNTM1, however only mildly sensitizes *MLL*‐rearranged ALL cells toward conventional chemotherapeutics.

### 3.3 Combinatorial high‐throughput screening of decitabine with other drug classes

**FIGURE 4:**
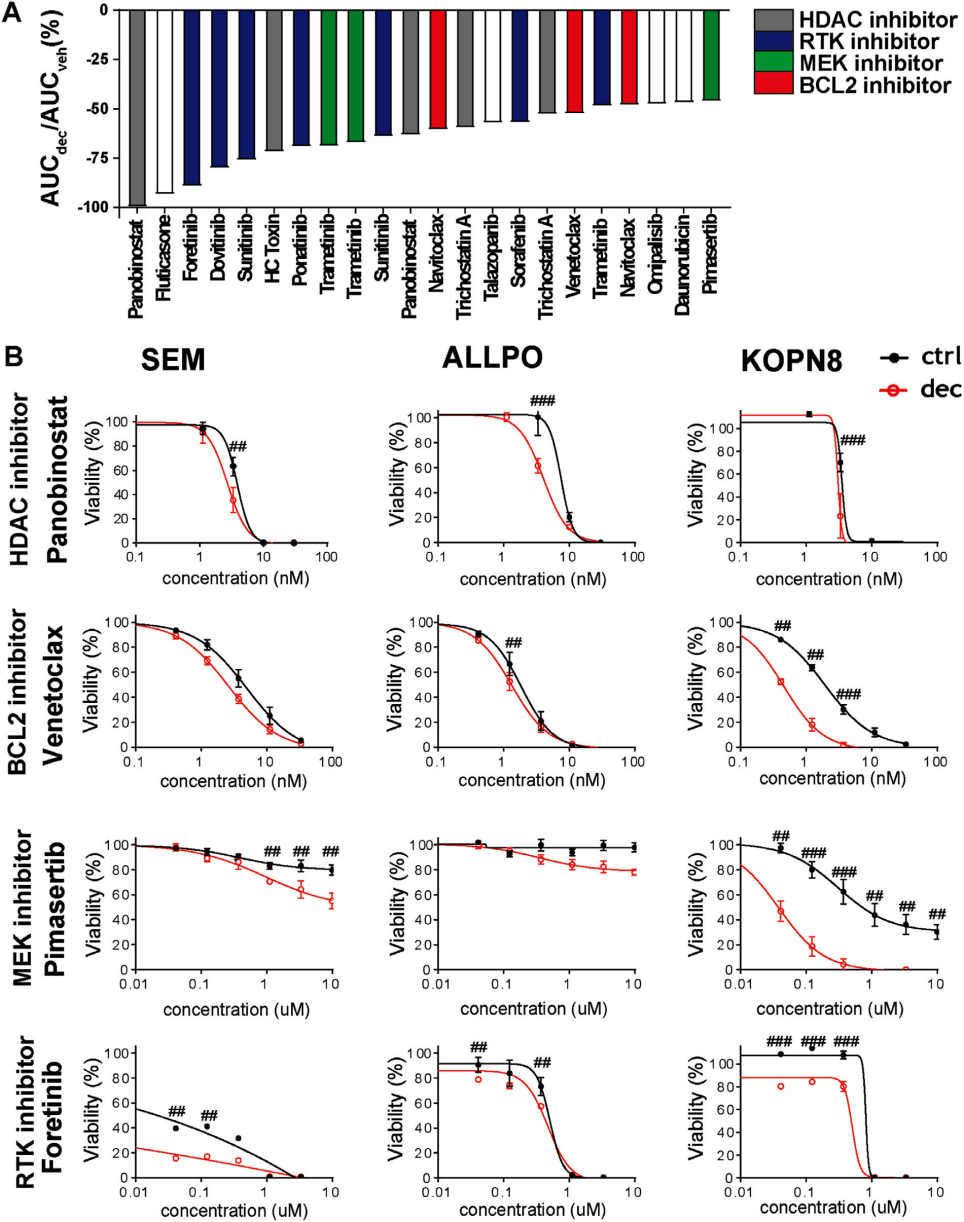
Long‐term low‐dose decitabine treatment fails to sensitize *MLL*‐rearranged ALL cells to epigenetic‐based or anti‐neoplastic agents. Overview top hits of drug library screens after 14 days low‐dose decitabine treatment in cell line SEM. Top hits were defined as drugs with a reduction of more than 30% of the area under the curve (AUC) in the decitabine pretreated SEM cells compared to AUC in the vehicle‐treated cells (A). Validation the drug screening top hits with an extended concentration range in the *MLL*‐rearranged leukemic cell lines SEM, ALLPO, and KOPN8. Decitabine pretreatment mildly sensitizes to the HDAC inhibitor Panobinostat, the BCL2 inhibitor Venetoclax, the MEK inhibitor Pimasertib, and the RTK inhibitor Foretinib. Synergy is determined using Bliss independence model. Percentage excess over Bliss (EoB) is indicated: ^##^EoB > 10%; ^###^EoB > 20% (B). Error bars represent the standard error of the mean (SEM). Graphs represent the average of n = 3 independent experiments (n = 2 for KOPN8)

Next, we assessed whether a prolonged pretreatment of low‐dose decitabine could sensitize *MLL*‐rearranged ALL cells to other epigenetic‐based or anticancer drugs. For this, SEM cells were exposed to a slightly higher concentration of 10 nM decitabine (or vehicle) compared to earlier experiments, for a period of 14 days. Subsequently, the sensitivity of these cells toward 369 different compounds, derived from an epigenetic‐based drug library and an anti‐neoplasm drug library, was tested using 4‐day MTT assays with drug concentrations of 10, 100, or 1000 nM. Results of all inhibitors tested are listed in Table S2. Drug synergy was based on area under the curve (AUC) calculations. A cut‐off of ≥30% difference in AUC of drugs in the decitabine pretreated SEM cells compared to vehicle‐treated cells was applied to determine the top hits, which could be mainly categorized as either histone deacetylase (HDAC) inhibitors, receptor tyrosine kinase (RTK) inhibitors, MEK inhibitors, and BCL2 protein family inhibitors (Figure 4A). As HDAC, MEK, and BCL2 inhibitors have shown promising pre‐clinical efficacy against *MLL*‐rearranged ALL [15, 38, 39, 40], these drug classes are of particular interest. Hence, we proceeded to validate a potential synergistic combinatorial effect of these compounds with decitabine in an extended *MLL*‐rearranged ALL cell line panel.

For all three cell lines tested, a chemo‐sensitizing effect was observed toward the HDAC inhibitor panobinostat, yet only for the combination of 3.3 nM panobinostat with decitabine (Figure 4B), potentially due to the steep dose‐response curve panobinostat elicits on its own. The combination of the BCL‐2 inhibitor venetoclax and decitabine enhanced the efficacy of venetoclax in KOPN8 cells.

MEK inhibitors have previously been shown to be effective for the treatment of *MLL*‐rearranged infant ALL cells harboring *RAS*‐mutations [38]. The effect of *RAS* mutations is represented here by the cell line KOPN8, while both cell lines ALLPO and SEM are *RAS*‐wild‐type. Interestingly, in the *RAS*‐wild‐type cell line SEM treated with decitabine the MEK inhibitor pimasertib revealed a mild chemo‐sensitizing effect, while showing a more pronounced chemo‐sensitizing effect in decitabine‐treated *RAS*‐mutant KOPN8 cells.

The combination of the RTK inhibitor foretinib and decitabine decreased cell viability more potently than either drug alone and indicated moderate synergy according to the Bliss independence model in all three cell lines tested.

Taken together, we showed drug synergy by means of the Bliss Independence model appeared moderate and was evident at limited drug concentrations (Figure 4B). Therefore, these data show that prolonged DNA demethylation by decitabine hardly sensitizes *MLL*‐rearranged ALL cells to known epigenetic‐based or anti‐cancer drugs.

## 4 DISCUSSION

We previously demonstrated the efficacy of DNA demethylating agents, such as decitabine and zebularine, in eradicating *MLL*‐rearranged ALL cells in vitro [9, 10]. However, not much research has demonstrated the efficacy of DNA demethylating agents against *MLL*‐rearranged ALL in vivo, while clinical trials have already been conducted for other types of leukemia. For instance, decitabine shows promising results against acute myeloid leukemia (AML) in both children and adults [29, 41].

Recently, Roolf et al, reported that decitabine induced a significant delay of leukemic progression *in vivo* in mouse xenografts of the *MLL*‐rearranged ALL cell lines SEM and RS4;11, but could not eradicate the leukemia [42]. In line with that study, our present data also show a significant delay in leukemia progression induced by decitabine in xenografts of the *MLL*‐rearranged ALL cell line SEM, albeit modestly. There are, however, clear differences in the experimental design of both studies. Roolf and co‐workers treated their mice with 0.4 mg/kg decitabine daily for only four consecutive days, seven days after leukemia injection. In contrast, we treated our mice with only 0.1 mg/kg decitabine three times a week over a period of three weeks, starting treatment three days after tumor injection. Moreover, THU was co‐administered in the mice, which is known to elevate decitabine plasma levels up to 10‐fold [22, 23, 24, 25, 27]. We deliberately choose to use 0.1 mg/kg of decitabine, as low‐dose decitabine is sufficient to deplete *MLL*‐rearranged ALL cells from DNMT1 (and thus to induce DNA demethylation), and prevents aspecific drug effects such as diminished DNA polymerase functioning [37]. Yet, it seems evident that the promising inhibitory effects of DNA demethylating agents against *MLL*‐rearranged ALL cells in vitro, were not indicative for similarly promising results in in vivo mouse models. Possibly, the typical experimental setup of in vitro drug response assays, in which tumor cells are cultured for a fixed period of time in the presence of increasing drug concentrations, provides a plausible explanation. If, as we show here, low concentrations of decitabine are sufficient to completely deplete *MLL*‐rearranged ALL cells from functional DNMT1, higher concentrations of decitabine should not provide any additional effects, other than aspecific drug effects that are not related to DNA demethylation. Thus, increasing concentrations of decitabine may eradicate most *MLL*‐rearranged ALL cells in in vitro drug response curves, this might not be solely due to DNA demethylation. If so, this may suggest that *MLL*‐rearranged ALL cells are not necessarily depending very heavily on their aberrant promotor DNA methylation patterns to maintain leukemogenic potential as observed previously [8, 9].

Additionally, we investigated whether prolonged low‐dose decitabine pretreatment could chemo‐sensitize *MLL*‐rearranged ALL cells toward chemotherapeutics currently used in the treatment of this type of leukemia, as well as toward various other epigenetic or anti‐neoplastic compounds. Earlier findings revealed that short‐term exposure to high‐dose decitabine could synergize with cytarabine to eradicate *MLL*‐rearranged ALL cells in vitro [42], as well as with l‐asparaginase to enhance cytotoxicity in the pediatric T‐ALL [30]. Here, we demonstrate that prolonged exposure to low‐dose decitabine occasionally sensitizes *MLL*‐rearranged ALL cells to some of the current chemotherapeutics at certain concentrations in some of the cell lines tested. These observations were most notable for l‐asparaginase and cytarabine, thereby confirming the results reported by others [30, 42].

Interestingly, the combination of decitabine and the MEK inhibitor pimasertib strongly decreased cell viability in *RAS*‐mutant KOPN8 cells than either drug alone. Previously, we showed that MEK inhibitors are effective for the treatment of *RAS*‐mutant *MLL*‐rearranged infant ALL cells [38, 39]. *RAS* mutations are found in 14‐24% of infant ALL patients and these *RAS* mutations decrease the survival chances even further [6]. Here, the MEK inhibitor pimasertib revealed a mild chemo‐sensitizing effect in the *RAS*‐wildtype cell line SEM, while showing a more pronounced chemo‐sensitizing effect in decitabine treated *RAS*‐mutant KOPN8 cells. Therefore, there might be a benefit for the treatment of *MLL*‐rearranged infant ALL harboring RAS mutations by combining decitabine and MEK inhibitors.

However, the in vitro chemo‐sensitizing effects of decitabine are modest and therefore clinical relevance may be rather limited. Additionally, since synergy was observed for limited concentration ranges, reaching and maintaining these exact concentration ranges in patients would be challenging due the many factors influencing the pharmacokinetics of the drugs [43, 44, 45, 46].

Furthermore, a recent study in relapsed pediatric ALL, all above 1 year of age, revealed that the combination of decitabine and HDAC inhibitor vorinostat in the current intensive chemotherapy protocol was determined not feasible due to the high incidence of infectious toxicities, despite encouraging response rates and pharmacodynamics [47]. The feasibility and efficacy of demethylating agents for the treatment of *MLL*‐rearranged infant ALL will be further evaluated in currrent clinical trials.

In conclusion, our present study demonstrates that prolonged exposure to a clinically relevant low‐dose of the DNA methyltransferase inhibitor decitabine significantly, but mildly delays leukemia progression in *MLL*‐rearranged ALL xenograft mouse models. Moreover, long‐term pretreatment with low‐dose decitabine moderately sensitizes *MLL*‐rearranged ALL cells toward conventional chemotherapeutics as well as toward known epigenetic‐based compounds and anti‐neoplastic agents, in vitro.

For a better understanding of the potential of demethylating agents in the treatment of *MLL*‐rearranged ALL, agents with increased stability and bioavailability could be further evaluated. Ongoing clinical trials should shed more light on theefficacy of demethylating agents for the treatment of *MLL*‐rearranged infant ALL.

## AUTHOR CONTRIBUTIONS

P.S., P.G.C., and R.W.S. designed the study; R.W.S. contributed materials; P.S., P.G.C, S.M.P., M.K., E.H.R. A.H.W.E., and M.E.M.D. performed the experiments; P.S., P.G.C., S.M.P., and M.K analyzed data; P.S. and P.G.C. performed statistical analysis; P.S., P.G.C., and R.W.S. wrote the paper; P.S., P.G.C., J.J.M., R.P., and R.W.S. supervised the study; and all co‐authors performed critical review of the manuscript and gave their final approval.

## STATEMENT ON ETHICS

Animal experiments were approved by the institutional Animal Ethical Committee of the Erasmus MC and performed in accordance to Dutch legislation.

## CONFLICT OF INTEREST

The authors declare that no conflict of interest exists.

## Supporting information

Supporting Information

Supporting Information

Supporting Information
