# Supplementary material for: Decitabine mildly attenuates MLL‐rearranged acute lymphoblastic leukemia in vivo, and represents a poor chemo‐sensitizer
Source: EJHaem. 2020 Aug 24;1(2):527–36. doi: 10.1002/jha2.81 (PMC9175850; doi:10.1002/jha2.81)
Supplement: Supplementary file 1 — Supporting Information [file JHA2-1-527-s002.pdf]

Supplementary Figure S1

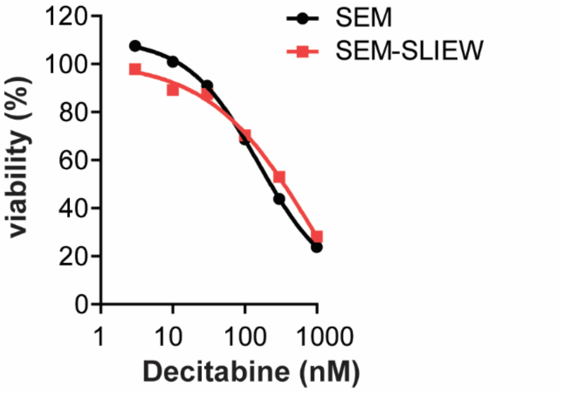

Figure S1: Dose response curve of SEM-SLIEW and the parental cell line SEM for decitabine

The *MLL*-rearranged ALL cell line SEM and its offspring, SEM-SLIEW, which has been modified to express eGFP and luciferase, were exposed for 4 days to a concentration range of decitabine. Viability was determined using MTS assay.
